# Comparison between AH plus sealer and total fill bioceramic sealer performance in previously untreated and retreatment cases of maxillary incisors with large-sized periapical lesion: a randomized controlled trial

**DOI:** 10.1038/s41405-024-00256-x

**Published:** 2024-09-12

**Authors:** Eisa Wahbi, Hassan Achour, Yasser Alsayed Tolibah

**Affiliations:** 1 Department of Endodontics, Faculty of Dentistry, Damascus University, P.O. Box 3062 Damascus, Syria; 2 Department of Pediatric Dentistry, Faculty of Dentistry, Damascus University, Damascus, Syria

**Keywords:** Diseases, Dentistry

## Abstract

**Objective:**

This study aims to assess the efficacy of bioceramic (BC) sealer when applied using the single cone technique (SCT), in comparison to AH Plus sealer applied with the cold lateral condensation technique (LCT), concerning their impact on the healing of large-sized periapical lesions in both untreated and retreatment cases.

**Materials and methods:**

A randomized controlled trial was conducted on 41 patients who had 60 permanent teeth in the maxillary incisors that were accompanied by large-sized periapical lesions. The included teeth were radiographed with cone beam computed tomography (CBCT) images and divided equally into two groups according to the endodontic treatment statute (30 untreated previous cases and 30 retreated cases). Subsequently, teeth received uniform preparation, irrigation, and dressing procedures. Then each group was divided equally according to the used sealer (15 teeth obturated using AH plus sealer with LCT and 15 teeth obturated using BC sealer with SCT). All cases were followed up after 6 and 12 months with CBCT images. Subsequently, the change in periapical lesion diameter (PLD) was observed as an indicator to determine the treatment outcome score. One-way ANOVA and Mann–Whitney U test were used to analyze the PLD changing and scoring the treatment outcome during the follow-up periods. The significance level was set at α = 0.05.

**Results:**

There were no statistically significant differences between untreated and retreatment cases neither using AH plus sealer with LCT nor BC sealer with SCT regarding changes in PLD in the follow-up periods. Moreover, there were no significant differences between groups regarding treatment outcome scores in the follow-up periods. All groups showed a high success rate.

**Conclusions:**

Successful treatment was achieved for untreated or retreatment maxillary incisor cases accompanied by large-sized periapical lesions using either BC sealer with the SCT or AH Plus sealer with LCT with a similar high success rate up to 12 months follow-up period.

## Introduction

Recent research in endodontic treatment highlights the significant role of microorganisms in initiating and perpetuating periapical diseases [1, 2].

The American Association of Endodontists has established a diagnostic classification for periapical diseases based on clinical and radiographic findings, encompassing a range of conditions including normal periapical tissues, characterized by healthy surrounding tissues and uniform periodontal ligament space with an intact lamina dura; symptomatic apical periodontitis, identified by painful response to biting and percussion, possibly with radiographic periapical translucency; asymptomatic apical periodontitis, which appears as a radiolucent area around the root without clinical symptoms; acute apical abscess, marked by rapid onset, spontaneous pain, tooth tenderness, purulent formation, and associated mucosal swelling; chronic apical abscess, distinguished by gradual onset, intermittent pus discharge through sinus tracts, and minimal or no discomfort; and condensing osteitis, seen as a radiopaque lesion at the root apex, indicating a bone reaction to low-grade inflammatory stimulus, typically from long-standing pulpal disease, while excluding commonly diagnosed conditions such as granulomas and cysts due to their histopathological basis rather than clinical or radiographic diagnosis [3–5].

Studies have shown that 58.6% of root canal treatment failures can be attributed to incomplete filling of the root canal system [6]. Therefore, the primary goal of successful root canal treatment is to prevent the transfer of microbes and their byproducts into the root canal system, reaching the periapical tissues. This is achieved through three-dimensional obturation of the root canal system and ensuring a hermetic seal in the coronal, apical, and lateral directions [7]. Proper sealing prevents any communication between the root canal and the periapical tissues, effectively blocking the spread of toxins and microbial products, thus preventing treatment failure [8, 9].

Effective apical sealing can be enhanced by using root canal sealers that chemically bond to the dentinal walls. Ideal sealers should possess qualities such as tight apical sealing, antimicrobial efficacy, resistance to dissolution in body fluids, chemical bonding to dentinal walls, and dimensional stability [10].

AH Plus sealer, an epoxy resin-based sealer, is considered the gold standard among root canal sealers due to its excellent physical, chemical, and biological properties [11, 12]. However, the biocompatibility of this sealer remains a subject of debate, as some studies raise concerns about potential inflammatory responses or cytotoxic effects associated with resin-based sealers [13].

Recent advancements have introduced bioceramic-based sealers composed of zirconium oxide, calcium silicates, calcium phosphates, and calcium hydroxide. These sealers are available in pre-mixed syringes for easy application and exhibit hydrophilic properties, allowing for setting reactions in a moist environment within the root canal, thus preventing shrinkage [14, 15]. Moreover, bioceramic-based sealers exhibit favorable biocompatibility due to their similarity to dentin and ability to stimulate mineralization [16].

The Lateral condensation technique (LCT) remains the most common obturation technique due to its simplicity, minimal equipment requirements, and ease of learning. Several studies have demonstrated that the sealing ability of the CLC technique is comparable to newer obturation techniques [17].

Modern trends in root canal obturation have shifted towards the use of the single cone technique (SCT). A recent study revealed that employing the single cone technique for root canal obturation with bioceramic sealers reduces the occurrence of procedural errors [18]. It is noteworthy that in a retrospective study conducted by Elizabeth A. Chybowski and colleagues in 2019 [19], it was found that teeth treated with the single cone and bioceramic sealer technique achieved a success rate of 90.9%, with the highest success rate observed in lesions with a diameter of less than 5 mm.

It’s crucial for dental professionals to carefully consider the biocompatibility aspect when selecting root canal sealers, taking into account factors such as clinical outcomes, patient health, and long-term effects on periapical tissues. Further research and clinical studies are needed to establish a clearer understanding of the biocompatibility profile of various root canal sealers and their impact on treatment outcomes and patient well-being.

In previous studies [20, 21], two-dimensional imaging was used to evaluate the use of calcium silicate-based and resin-based sealers with standardized obturation techniques (lateral compaction) on samples that might not have been strictly uniform, to study the effect of sealer type on the healing of periapical lesions. However, to the best of our knowledge, this study is one of the first randomized clinical trials to focus on changing the obturation technique and sealer type to assess their impact on the healing of large-sized periapical lesions resulting from necrotic pulp or previously unsuccessful endodontic treatment, using a controlled sample of maxillary anterior teeth evaluated with three-dimensional imaging.

## Materials and methods

### Study design, settings, and ethical approval

This randomized double-blinded controlled trial has utilized a two-arm parallel superiority design. This study was conducted from January 2020 and January 2023 at the Endodontic Department Faculty of Dentistry, Damascus University, Damascus, Syria. This study was conducted respecting the ethical guidelines of the Declaration of Helsinki. The research project was ethically approved by Damascus University (approve number: UDDS-2038-22062020/SRC-414). The project was funded by Damascus University (funder No. 501100020595) and it was retrospectively registered at clinical trials.gov under ID number: NCT06400030. This RCT has been written according to CONSORT 2010 guidelines.

### Sample size calculation

The sample size was calculated using G* Power 3.1.9.4 (Heinrich-Heine-Universität, Düsseldorf, Germany). It was estimated depending on a previous study [22], which described the changes in periapical lesion diameter using CBCT images after 12 months of endodontic treatment. A minimum total sample size of 60 patients (15 in each group) was found to be sufficient for a level of significance of 0.05, power of 80%, and 0.45 as effect size f.

### Recruitment and eligibility criteria

**Fig. 1:**
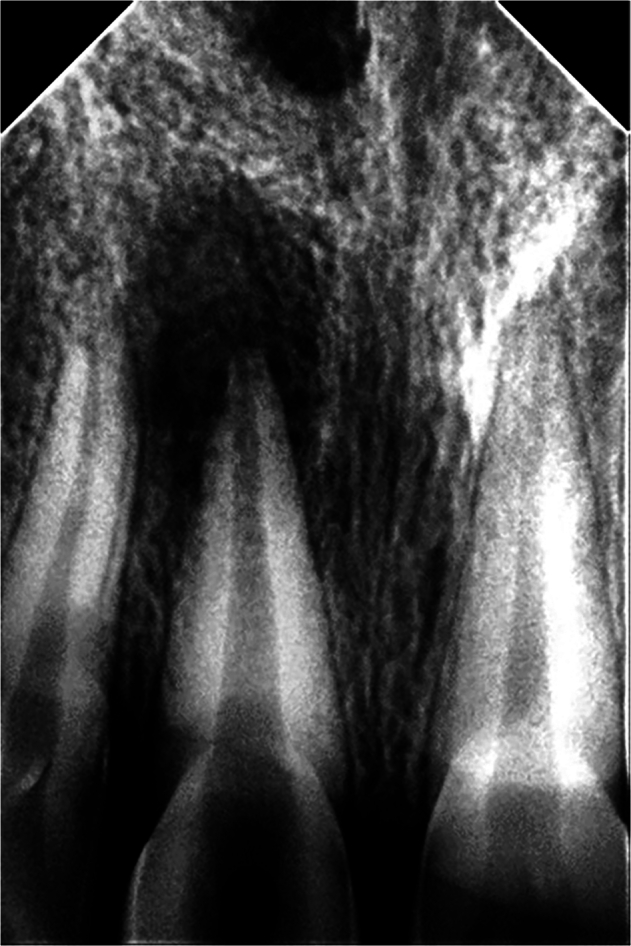
A diagnostic periapical radiograph of large periapical lesion on the maxillary central incisor.

One hundred and thirty patients aged between 18 and 40 years were referred to the Endodontic Department during the study period because the presence of apical lesions in their teeth was investigated by the principal research (I.W). The principal investigator conducted a search for patients with one or more maxillary anterior teeth accompanied by periapical lesions requiring root canal treatment (necrotic pulp) or retreatment (failed previous endodontic treatment) exhibiting large-sized periapical lesions greater than 5 mm (classified as S3 according to the Venskutonis classification) [23]. Preoperative periapical radiographs (Fig. 1) were captured to estimate the anatomy of the incisors and the size of the periapical lesion, the diameter of the apex, and the etiology of the periapical lesion in order to identify the included incisors. Those who met this condition were one hundred patients. Fifty-nine patients were considered unsuitable for inclusion due to the presence of general systemic diseases, un-restorable incisors, teeth with acute periapical abscesses, multi-infected teeth with interconnected periapical lesions, patients with advanced periodontitis (more than 5 mm periodontal attachment and bone loss), open-apex incisors, incisors with multi-canals, internal or external resorptions, curved-canals incisors, and the presence of cracks or fractures in the incisors.

Therefore, forty-one patients with 60 permanent incisors (30 teeth without previous endodontic treatment, and 30 teeth with previous endodontic treatment) were included in the current research. All included patients, who accepted to participate in this study, signed an informed consent sheet after explaining all the details about the trial and the therapeutic part of it.

### Randomization

Incisors were assigned to control group (This group received obturation using AH Plus resin-based sealer and lateral condensation technique group), and Study group (This group received obturation using Total Fill BC Sealer bioceramic-based sealer and single cone technique). With 1:1 allocation ratio, a random sequence was created using the website www.random.org, which was accessed on 20 September 2020. Thus, patients were assigned to 4 groups: Group 1: AH Plus resin-based sealer in previously untreated cases = 15, Group 2: AH Plus resin-based sealer in retreatment cases = 15, Group 3: Total Fill BC Sealer in previously untreated cases = 15, and Group 4: Total Fill BC Sealer in retreatment cases = 15.

### Blinding

In the current study, a double-blinded approach was implemented, involving both patients and assessors. While the treating clinician could not be blinded due to the interventional nature of the study and their involvement in treatment, the patients were unaware of the specific sealer used during the procedure. Moreover, evaluation of treatment outcomes was carried out by two trained researchers (Ph.D. students) who were calibrated to the assessment criteria and kept unaware of the sealer used in each case.

### Clinical procedure

**Fig. 2:**
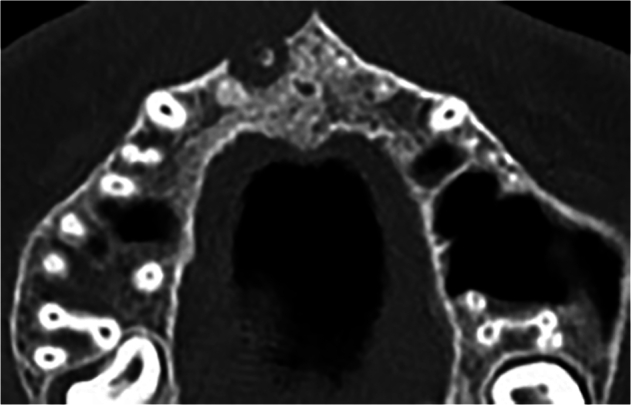
Preoperative CBCT image of the maxillary central incisor in axial view.

The patient was requested to undergo cone-beam computed tomography (CBCT) imaging of the maxillary anterior region before starting the treatment to assess the dimensions of the periapical lesion around the apex, as depicted in Fig. 2.

The root canal treatment or retreatment was performed by a single specialist in endodontics in two sessions (Two-Visit). After applying local anesthesia using 2% lidocaine with 1:80,000 epinephrine, the treated tooth was isolated with a rubber dam, and any previous restoration was removed if present. Decay was thoroughly excavated, then the pulp chamber was accessed using an Endo-Z bur.

#### Treatment cases (necrotic pulps, without previous endodontic treatment)

Initially, canal patency was confirmed using #10 and #15 K-files (Mani, Utsunomiya, Japan). Then, preparation was initiated with an SX file of Protaber system (Protaper, Dentsply Maillefer, Ballaigues, Switzerland) after setting the rotary device (X-Smart, Dentsply, Switzerland) to a speed of 300 rpm and torque of 3 N.cm, until resistance was felt during rotation. Afterward, working length was determined using an apex locator with a K-file size #10 and confirmed radially. Then, S1 and S2 files were used to reach the full determined working length. Preparation was completed using finishing files (F1, F2, and F3) to the full determined working length. Canal patency was ensured between each file and the following one using a K-file size #10 or #15, with irrigation (2 ml) of 5.25% sodium hypochlorite solution between used files [24]. Additionally, 17% EDTA gel was applied as a lubricant on each file before insertion into the root canal.

#### Retreatment cases (failed previous endodontic treatment)

The previous filling within the root canal system was removed using the ProTaper Universal Retreatment system, where the rotary was at set a speed of 500 rpm and torque of 2.6 N.cm. Initially, entry was made with a D1 file (size 30, taper 0.09) to utilize its active tip in removing gutta-percha from the coronal third of the canal. Then, 1 ml of a solving agent (K-dental SKU, USA) was placed to soften the previous filling material. Subsequently, D2 file (size 25, taper 0.08) was used for the middle third, and D3 file (size 20, taper 0.07) for the apical third, after determining the working length using an apex locator with a K-file size #10 and radially confirming. Preparation was completed using finishing file F3 to the full determined working length. Canal patency was ensured between each file and the following one using a K-file size #10 or #15, with irrigation (2 ml) of 5.25% sodium hypochlorite solution between used files. Additionally, 17% EDTA gel was applied as a lubricant on each file before insertion into the root canal. Smoothness of the root canal walls and absence of gutta-percha residue on the last used file indicate completion of preparation in these teeth [25, 26].

#### Root canal dressing application

Regardless of whether the tooth had treatment or retreatment, root canal dressing was applied after finishing the chemomechanical preparation of the root canals. Calcium hydroxide paste (Metapex, Meta, South Korea) was applied for a week. Then, the teeth were sealed using a temporary restoration of chemically cured glass ionomer cement (GIC, Shanghai Rong Xiang Dental Material Company, Ltd, China), ensuring tight sealing of the access cavity to prevent any coronal microleakage. The patient was instructed to return for the second appointment after one week.

#### Removal of root canal dressing and final irrigation protocol

After anesthesia, isolation with a rubber dam, and removal of the temporary restoration, the root canal dressing was removed by performing circumferential filing of the canal walls using an H-file, with irrigation using physiological saline solution, followed by final irrigation with 5.25% sodium hypochlorite solution for 40 s, 17% EDTA solution for 60 s, another irrigation with 5.25% sodium hypochlorite solution for 40 s, 2% chlorhexidine solution, and ensuring irrigation with distilled water, alternated between the previous irrigation solutions. Finally, the root canals drying with paper points was conducted [27].

#### Root canal obturation

The master cone (F3) was selected for its ability to reach the full working length and exhibit slight resistance at the apex (Tug-Back), and underwent radiographic confirmation.

##### Control group

The AH Plus sealer’s base and accelerator ((Dentsply Sirona, Charlotte, NC, USA)) were mixed. Subsequently, the canal walls were coated with sealer using a K-File in a determined counterclockwise motion. The master cone (F3) was generously coated with sealer and inserted into the root canal until reaching the full working length. Lateral condensation was performed, where the spreader was placed into its maximum depth, and then it was removed by rotating it back and forth as it was withdrawn. The accessory cones (25.02, and 20.02) were placed in the space vacated by the spreader. The process was repeated until the spreader could be inserted no more than 2 mm from the canal orifice [28].

##### Study group

After partially drying the root canals, the BC sealer-loaded syringe (Totalfill BC Sealer, FKG Dentaire SA, La Chaux-de-Fonds, Switzerland) was inserted into the root canal up to the coronal third, administering the sealer slowly while retracting the syringe, ensuring synchronous withdrawal. The sealer-coated cone was delicately placed into the root canal to the predetermined working length without exerting undue pressure [29].

**Fig. 3:**
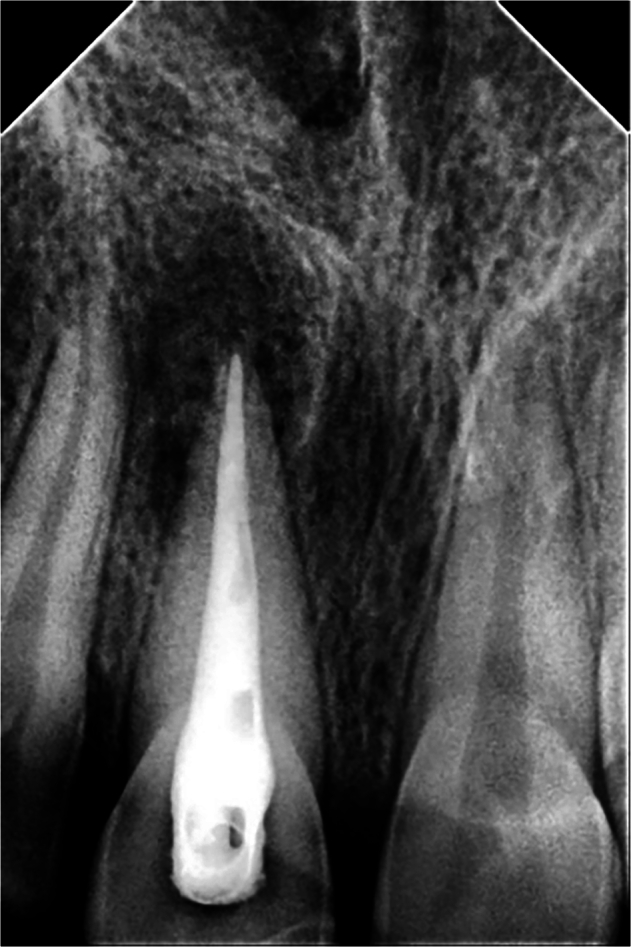
Post-operative periapical radiograph to ensure the obturation quality.

In both groups, periapical radiographs were taken to ensure the obturation quality (Fig. 3).

Following the obturation procedure in both groups, any excess sealer or gutta-percha were removed and a periapical radiograph was taken to validate the quality of the root canal filling. A base filling of glass ionomer cement was applied, succeeded by the definitive restoration using composite resin restorations (Tetric N-Ceram, VivaDent), checking occlusion, and executing thorough finishing and polishing techniques.

### Follow-up

**Fig. 4:**
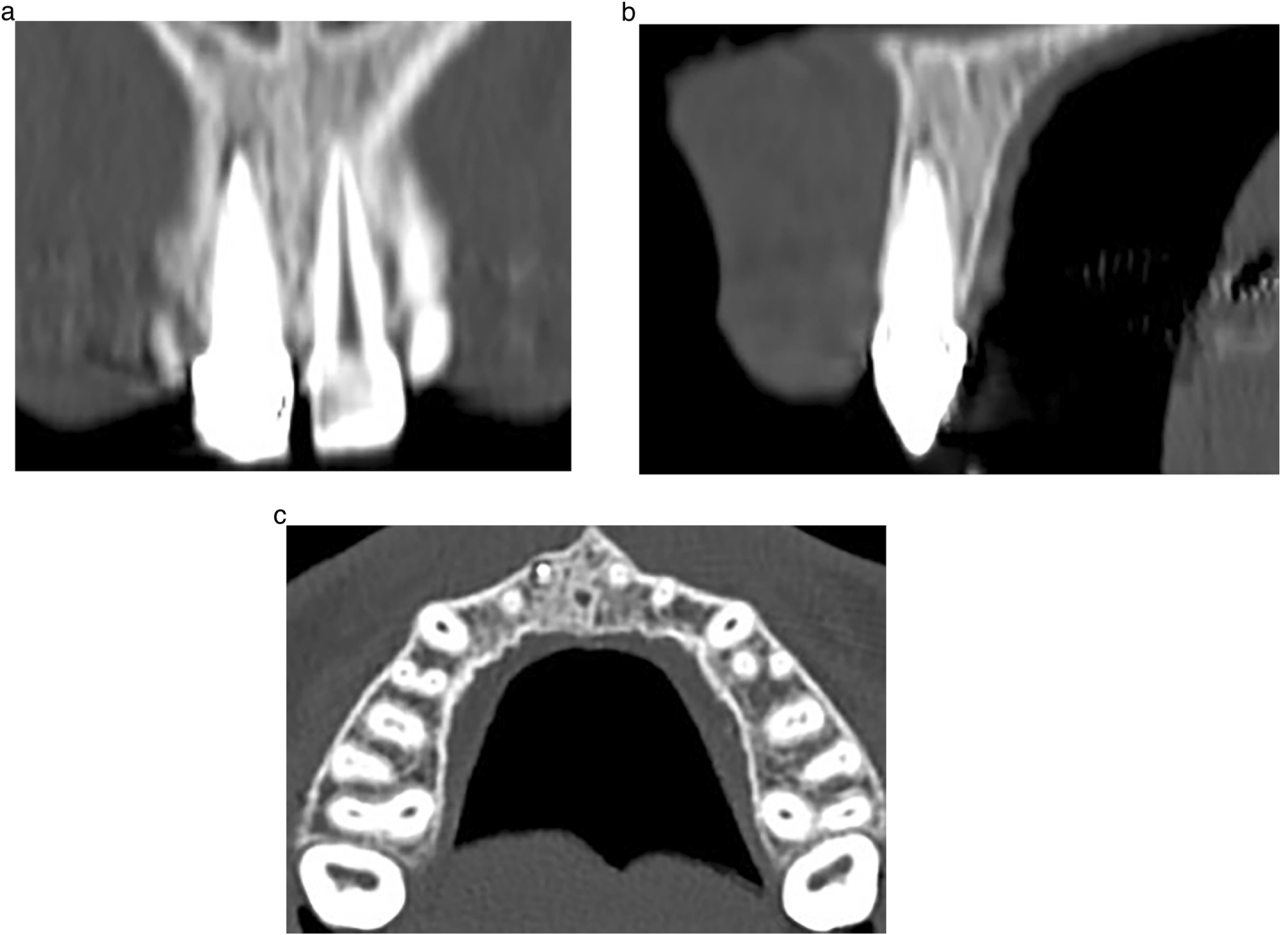
The first radiographical follow- up views. CBCT image after 6 months of treatment; **a**: Coronal view, **b**: Sagittal view, and **c**: Axial view.

**Fig. 5:**
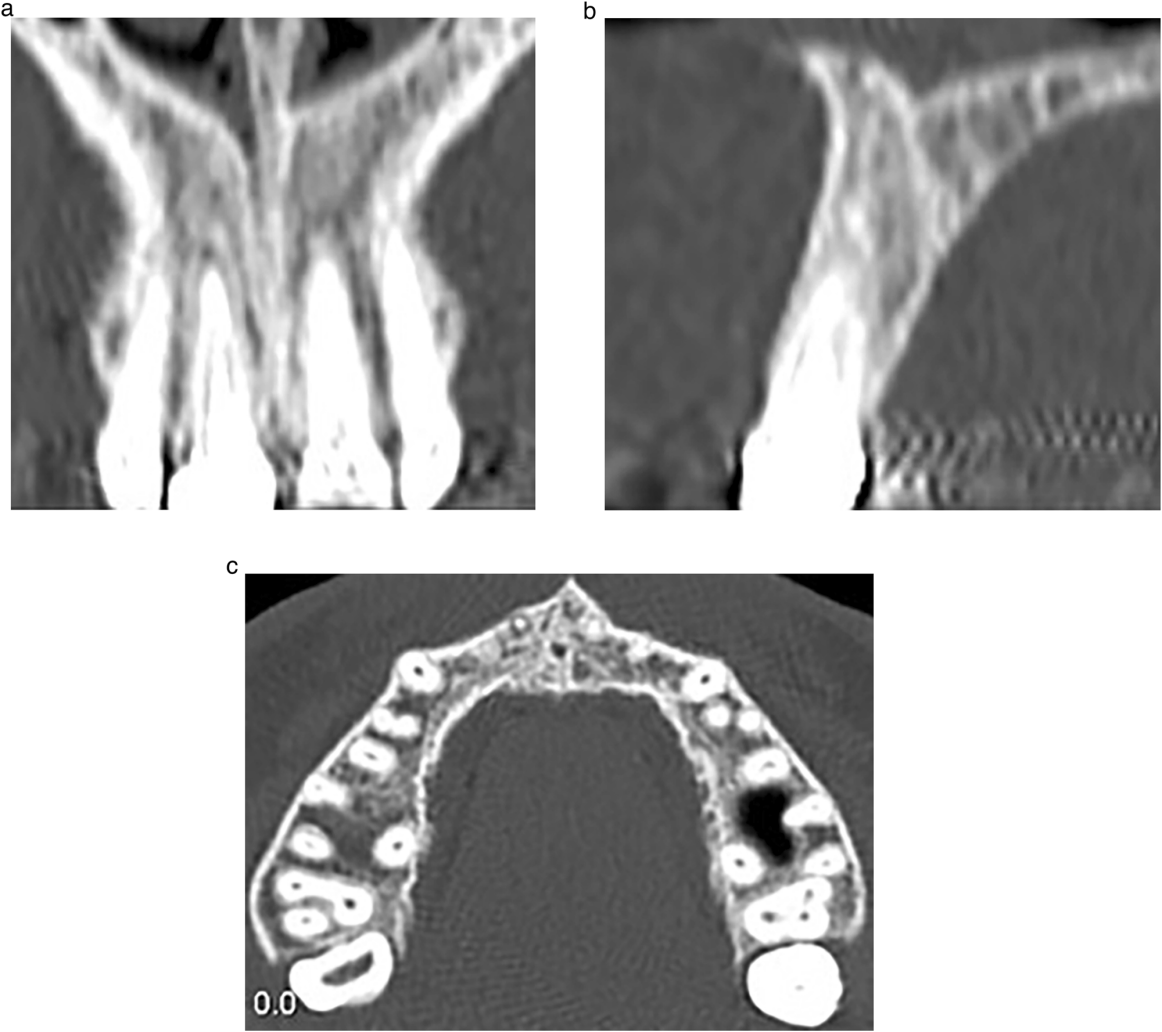
The second radiographical follow- up views. CBCT image after 12 months of treatment; **a**: Coronal view, **b**: Sagittal view, and **c**: Axial view.

The periapical lesions were evaluated by cone-beam computed tomography (CBCT) radiographs that were taken for each patient at three time points: before the treatment (Fig. 2), 6 months after the treatment (Fig. 4), and 12 months after the treatment (Fig. 5).

The CBCT imaging standards for the maxillary anterior region were uniformly adopted in the three images, where the patient’s face was ensured to be parallel to the horizontal plane aligning the longitudinal axis with the midline of the face, and the transverse axis with the corners of the eyes. The radiology technician ensures that acquired sections adhere to specific orientation rules: sections in the sagittal plane were parallel to the longitudinal axis of the tooth both horizontally and vertically, while in the horizontal plane, they were parallel palatally and buccally, and in the axial plane, they were perpendicular to the longitudinal axis of the tooth.

The three CBCT images were at a setting of 85 Kvp voltage, 9 mA milliamperage, 14 S exposure time, 10 × 15 cm field of view and 0.2 mm voxel size.

### Outcomes measurement

**Fig. 6:**
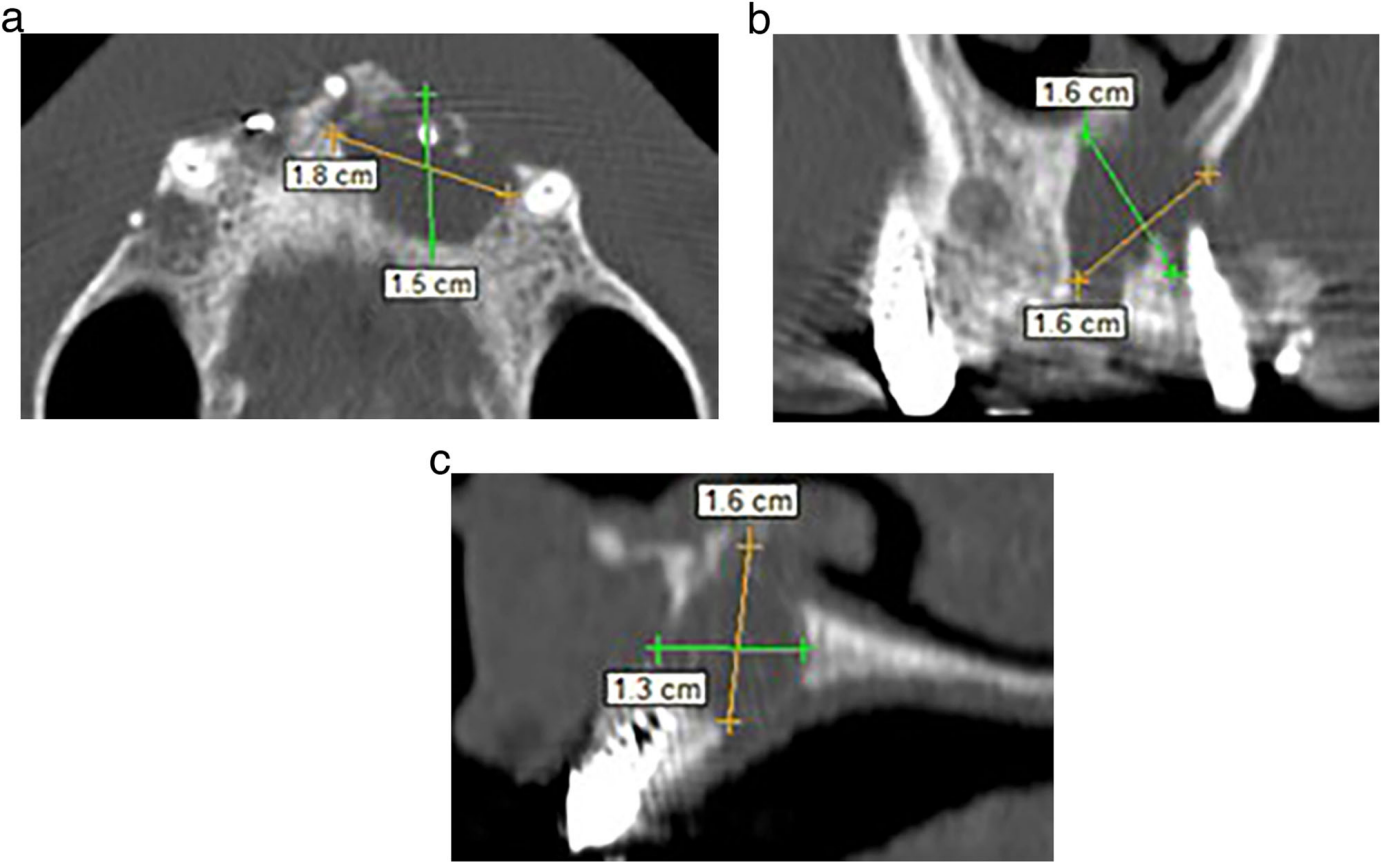
Outcomes measurement procedures. Determining the apical lesion diameter in CBCT image sections; **a**: in the Axial section, **b**: in the Coronal section, and **c**: in the Sagittal section.

Each incisor’s periapical lesion was estimated before treatment and after 6 and 12 months of treatment by measuring the periapical lesion diameter (PLD) in millimeters in the three planes (sagittal, horizontal, and axial) using the scale provided in the CBCT image program, then calculating the mean. The following rules were followed: Initially, the acquired sections of the lesion on the CBCT image were studied in the three planes, and the section containing the largest area of the apical lesion in each studied plane was selected. If the shape of the apical lesion is symmetrically circular in one of the three planes, the PLD in that plane was measured using the scale in the CBCT image program. However, if the shape of the apical lesion is non-circular in one of the three planes, the largest and smallest diameters of the lesion in that plane were measured using the scale in the CBCT image program, then the mean diameter was calculated. Finally, the three diameters of the lesion (obtained in the three planes) were summed and divided by 3 to obtain the mean diameter of the studied apical lesion (Fig. 6).

According to the PLD changing, the results of the monitored cases were categorized into four groups as a treatment outcome scores:

1. Healed: The periapical lesion completely disappeared in the radiograph.
2. Healing: The periapical lesion showed a reduction in the size of the lesion without complete disappearance in the radiograph.
3. Doubt: The periapical lesion size remained the same in the radiograph.
4. Failure: The periapical lesion size increased after treatment in the radiograph.

The previous procedure was done by two blinded trained researchers (Ph.D. students) who were calibrated to the assessment criteria and kept unaware of the sealer used in each case. Subsequently, a third trained researcher randomly reviewed 10% of the cases. The results from this third evaluation concurred with the initial one, as confirmed by Cohen’s κ test (K = 0.970, *p* ≤ 0.001).

### Statistical analysis

The data were analyzed using version 20 of the SPSS software, and the Kolmogorov–Smirnov test indicated that the data distribution was normal. Therefore, the One-way ANOVA test was used to compare the size of the periapical lesions among the groups during the follow-up periods and the Mann–Whitney U test was used to compare the treatment outcome scores among the groups during the follow-up periods. The level of significance was set at α = 0.05.

## Results

Sixty maxillary teeth with necrotic pulps were included from 41 patients (18 men and 23 women). Table 1 shows the distribution of patients by age within the study groups. No statistically significant differences were recorded between the groups regarding age (*P* = 0.754) for the treated patients. Table 2 hows the distribution of patients by gender within the study groups. No statistically significant differences were recorded between the groups regarding gender (*P* = 0.965) for the treated patients.

**Table 1:** Descriptive and analytic statistics of age distribution across groups.

Group	Mean	Standard deviation	Minimum	Maximum	*P*value^a^
AH Plus resin-based sealer in untreated cases	22.65	4.789	19	35	0.754
AH Plus resin-based sealer in retreatment cases	21.80	5.088	18	40	
Total Fill BC Sealer in untreated cases	23.35	5.434	20	40	
Total Fill BC Sealer in retreatment cases	22.25	5.566	18	38	

^a^One-Way ANOVA.

**Table 2:** Descriptive and analytic statistics of distribution of included incisors by gender of the patients.

Group	Sex		Total	*P*-value^a^
	Male	Female		
AH Plus resin-based sealer in untreated cases	7 (46.67%)	8 (53.33%)	15 (100%)	0.965
Total Fill BC Sealer in untreated cases	6 (40%)	9 (60%)	15 (100%)	
AH Plus resin-based sealer in retreatment cases	6 (40%)	9 (60%)	15 (100%)	
Total Fill BC Sealer in untreated cases	7 (46.67%)	8 (53.33%)	15 (100%)	

^a^Chi-square test.

The second table summarizes the distribution of included incisors by gender of the patients. No statistically significant differences were recorded between the groups regarding gender (*P* = 0.965) for the treated patients.

**Fig. 7:**
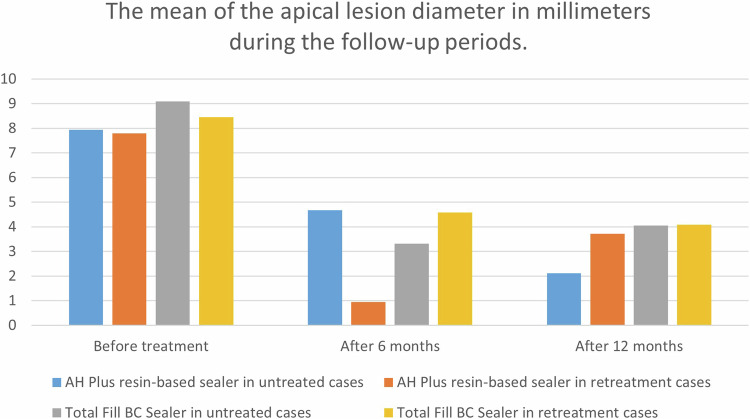
A diagram of the mean of the apical lesion diameter in millimeters during the follow-up periods.

Table 3 summarizes the mean (Fig. 7), the standard deviation, the range, and the One-way ANOVA test results of the apical lesion diameter in millimeters before treatment, 6 months, and 12 months of treatment in the groups. The One-way ANOVA test showed no significant differences between the groups (AH Plus resin-based sealer in treatment cases, AH Plus resin-based sealer in retreatment cases, Total Fill BC Sealer in treatment cases, and Total Fill BC Sealer in retreatment cases) before treatment, 6 months, and 12 months of treatment regarding the apical lesion diameter in millimeters (*P* = 0.172, *P* = 0.387, and *P* = 0.176 respectively).

**Table 3:** The mean, the standard deviation, the range, and the One-way ANOVA test results of the apical lesion diameter in millimeters during the follow-up periods.

Studied period	Group	Number	Mean ± Std. deviation	Range	*F*-value	*P*-value^a^
Before treatment	AH Plus resin-based sealer in untreated cases	15	7.94 ± 2.01	5–11	1.382	0.172
	AH Plus resin-based sealer in retreatment cases	15	7.79 ± 3.12	5–14		
	Total Fill BC Sealer in untreated cases	15	9.10 ± 2.51	5–14		
	Total Fill BC Sealer in retreatment cases	15	8.46 ± 3.97	5–18		
After 6 months	AH Plus resin-based sealer in untreated cases	15	4.67 ± 2.35	2–10	0.872	0.387
	AH Plus resin-based sealer in retreatment cases	15	0.95 ± 3.55	0–14		
	Total Fill BC Sealer in untreated cases	15	3.31 ± 0.74	0–14		
	Total Fill BC Sealer in retreatment cases	15	4.58 ± 1.27	0–14		
After 12 months	AH Plus resin-based sealer in untreated cases	15	2.11 ± 2.61	0–16	1.369	0.176
	AH Plus resin-based sealer in retreatment cases	15	3.71 ± 4.18	0–14		
	Total Fill BC Sealer in untreated cases	15	4.05 ± 3.65	0–16		
	Total Fill BC Sealer in retreatment cases	15	4.08 ± 4.42	0–7		

^a^One-way ANOVA test.

Table 4 presents the results of treatment outcomes in the research sample based on the PLD changing among the four groups during the follow-up periods and Mann–Whitney U test results.

**Table 4:** Assessing the treatment outcomes in the research sample based on the change in the diameter of the periapical lesion among the groups and Mann–Whitney U test results during the follow-up periods.

Studied period	Group	Number	Failed	Doubt	Healing	Healed	U-value	*P*-value^a^
After 6 months	AH Plus resin-based sealer in untreated cases	15	1 6.67%	1 6.67%	13 86.67%	0 0%	512.5	0.735
	AH Plus resin-based sealer in retreatment cases	15	0 0%	0 0%	14 93.33	1 6.67%		
	Total Fill BC Sealer in untreated cases	15	0 0%	1 6.67%	12 80%	2 13.33		
	Total Fill BC Sealer in retreatment cases	15	1 6.67%	1 6.67%	12 80%	1 6.67%		
After 12 months	AH Plus resin-based sealer in untreated cases	15	1 6.67%	0 0%	7 46.67%	7 46.67%	419.5	0.089
	AH Plus resin-based sealer in retreatment cases	15	0 0%	0 0%	9 60%	6 40%		
	Total Fill BC Sealer in untreated cases	15	1 6.67%	0 0%	11 73.33%	3 20%		
	Total Fill BC Sealer in retreatment cases	15	1 6.67%	0 0%	10 66.67%	4 26.67%		

^a^Mann–Whitney U test.

The Mann–Whitney U test revealed no significant differences in the success and failure rates between the four groups, indicating similar performance among the sealers used in both untreated cases and retreatment cases.

## Discussion

Periapical lesion treatment varies from conservative endodontic therapy with or without periapical surgery to tooth extraction. Since most periapical lesions are caused by an inflammatory reaction within the root canal system, a conservative endodontic approach should always be preferred over surgery [30].

Conventional root canal procedures use gutta-percha cones and sealers to ensure a tight seal. Endodontic sealers vary in their properties, and no single material has all the ideal qualities. AH Plus sealer is the standard because of its excellent physical and chemical properties, although it lacks bioactive potential [31–33].

AH Plus sealer is considered the standard sealer due to its excellent physical and chemical properties, despite lacking bioactive potential [34].

However, recently, calcium silicate-based sealers have emerged and garnered significant interest due to their desirable properties, including their ability to provide a good seal of the root canal when combined with one or more gutta-percha cones, antimicrobial activity, and their ability to adhere to the root canal walls [35]. Total Fill BC Sealer, a modern generation sealer, is capable of preventing apical leakage due to its bonding with dentin walls and gutta-percha cones [36].

A rigorous scientific methodology was followed in the current study to to evaluate the efficacy of the Totalfill BC Sealer in SCT compared to the AH Plus sealer in LCT by assessing their impact on large-sized periapical lesion healing in previously untreated and retreatment cases.

This clinical study was conducted on healthy patients without systemic diseases to exclude any systemic factor that could affect lesion healing. All included teeth were permanent maxillary incisors to standardize the preparation and obturation of the root canals, unify the factors affecting healing, and facilitate lesion healing monitoring. Moreover, both untreated or retreatment cases had large periapical aiming to evaluate the impact of previous endodontic treatment on the final treatment outcome.

Cases with large lesions were selected, as there is generally a noticeable decrease in the success rate of root canal treatment with larger periapical lesions (those with a diameter exceeding 5 mm), which may be attributed to the lower presence of osteoblast progenitors within large lesions, which are often cystic [37]. Another reason is that larger lesions may allow for a more accurate assessment of behavior and healing speed for both sealers used. It is noteworthy that multi-infected teeth with interconnected periapical lesions were excluded from this study to accurately determine the diameter of each lesion. Even if the lesion involved adjacent healthy teeth, these teeth remained unaffected and maintained their vitality throughout the follow-up period when the main lesion healed.

The ProTaper rotary preparation system was utilized in all included incisors, employing a unique concept comprising only 6 files: three shaping files (SX, S1, S2) and three finishing files (F1, F2, F3). The unique feature of shaping files is their multiple percentage taper along the working blades, enhancing flexibility, cutting efficiency, and safety. Each file shapes a specific area of the root canal, minimizing the risk of fatigue failure. In contrast to shaping files, finishing files have a reduced taper, improving flexibility and preventing excessive widening of the coronal and middle thirds of the canal [38].

The root canals were irrigated with 5.25% sodium hypochlorite, which is considered the most effective irrigant for root canal disinfection during root canal preparation procedures [39]. Sodium hypochlorite dissociates into hypochlorite ions (OCl^−^), sodium ions (Na+), and hypochlorous acid, which have strong antimicrobial effects, particularly against anaerobic bacteria such as E. faecalis, and directly dissolve necrotic tissue [40].

17% EDTA was used to remove the smear layer, enhancing the adaptation of root canal filling materials to dentin walls, followed by irrigation with saline solution [41].

This protocol is typically recommended for irrigation and cleaning to remove the smear layer. Chelating agents like EDTA remove only the inorganic components of the smear layer, leaving organic elements intact, necessitating the use of sodium hypochlorite solution after EDTA irrigation to eliminate it [27].

Since the study was conducted on necrotic pulp cases associated with periapical lesions, a 2% chlorhexidine (CHX) solution was applied at the end of the irrigation and cleaning phase. CHX is effective against a broad spectrum of microorganisms, particularly E. faecalis, and is less toxic [27].

Root canal treatment was performed over two visits, where calcium hydroxide dressing was applied in the first one. A study comparing single-visit and two-visit root canal treatment showed a decrease in periapical lesion size after six months in both groups, with no statistically significant differences observed between them [42]. In the current study, calcium hydroxide dressing was preferred due to the presence of several cases with clear clinical symptoms (sinus tracts, periapical swelling, suppuration) associated with large periapical lesions [43]. Many researchers believe that root canal treatment with calcium hydroxide dressing improves bone healing and regeneration [44]. However, others have shown that calcium hydroxide has no effective impact on all endodontic pathogens and microorganisms and thus does not contribute significantly when used as an inter-appointment dressing [45].

LCT was employed with AH plus sealer as it is considered the standard technique for comparison with other techniques, allowing for adaptation of the sealer material to root canal irregularities [46].

Modern trends in root canal obturation have shifted towards the SCT, as recent studies have revealed that using the SCT in root canal obturation with bioceramic sealers reduces procedural errors [18]. Moreover, the use of bioceramic sealers is recommended with the SCT, as heat can cause deterioration of their physical properties, resulting in decreased bond strength. Additionally, heat can lead to a reduction in setting time and flow rate [47].

It is worth noting that no case exhibited the extrusion of excessive materials (gutta-percha and sealers) beyond the apex. Therefore, this factor was not estimated in the current study, as the relationship between overfilling and periapical lesion healing remains controversial. While some studies have reported an association between failed endodontic treatment and overfilling [48], others have found no such correlation [49].

Cone-beam computed tomography (CBCT) imaging is more accurate than conventional radiography in evaluating periapical lesions, especially when the lesion size exceeds 1.4 mm [50, 51].

The cases in the current study were followed up at different time intervals (6 months, 12 months), as in many previous studies [52, 53]. A study by Penesis and colleagues demonstrated that a duration of 12 months may be optimal for assessing changes in bone density in the periapical area. This is because the decrease or regression in the size of the lesion, changes in bone density, and appearance of trabecular bone may serve as indicators of treatment success and healing of the periapical lesion, eliminating the need for further follow-up [54].

The results of the current study indicate that the entry of patients into the four study groups according to their ages, gender, and the diameter of the periapical lesions before treatment was homogeneous, with no statistically significant differences, indicating the presence of random distribution, which ensures the reliability of the observed results. Therefore, the effect of these variables was ignored as a previous study did [55].

The results of the current study concluded that there were no statistically significant differences in the success rates of root canal treatment or retreatment using both proposed sealers. Additionally, the success rates for both sealers were high with low failure rates.

Although bioceramic sealer possesses superior properties in apical sealing and biocompatibility compared to AH Plus sealer [56, 57], the antimicrobial capability of AH Plus against enterococcus faecalis bacteria was found to be greater than that of bioceramic sealer [58]. Moreover, both sealers exhibited bone healing properties without differences in their performance [59]. According to the observed results, the clinical performance of both sealers in healing large-sized periapical lesions in maxillary incisors was similar after standardizing all other factors.

The current results are consistent with the findings of Zavattini et al., who conducted a non-randomized clinical study to evaluate the success of endodontic treatment in necrotic or inflamed teeth. They standardized the root canal cleaning procedures and then obturated 51 root canals using the AH Plus sealer with the vertical compaction technique and 52 root canals using with BioRootTM RCS bioceramic sealer with single-cone obturation technique. They evaluated the success of endodontic treatment after 12 months using CBCT and periapical radiographs, and the success rate of the previous group t was 80-89% and 84-90% respectively with no statistically significant differences between the two groups [60].

Additionally, the results of our current study were similar to those of Jinghao and colleagues, who found that both the bioceramic sealer (iRoot SP) and AH Plus exhibited similar success rates after two years of follow-up when used on necrotic teeth or with irreversible pulpitis after employing a standardized preparation and irrigation protocol [22].

Similarly, Thakur et al. [20] found that using an MTA-based sealer can be comparable to a resin-based sealer in the healing of periapical lesions. However, this study included single-canal teeth from both the maxilla and mandible, all of which were obturated using the standardized cold lateral compaction technique, and the evaluation method was based on two-dimensional imaging.

The current findings met Santos-Junior and colleagues’ study, which demonstrated that the success rate of endodontic treatment for untreated teeth with necrotic pulp was 98.6%, while the success rate for retreatment cases was 95.6%. There were no statistically significant differences between the two groups. Moreover, this study revealed that the increased success rates of the treatments were attributed to improvements in the materials and tools used, as well as the increased use of magnification methods in the context of endodontic treatments [61].

In the study conducted by Khandelwal et al. [21], a randomized controlled clinical trial was performed to evaluate the effect of sealer type (resin-based vs. bioceramic-based) on the healing of teeth with necrotic pulp and periapical lesions. The findings indicated that the bioceramic-based sealer was superior. The discrepancies with the current study could be attributed to differences in follow-up periods. Additionally, this study standardized the obturation technique using cold lateral compaction for both groups and employed a different method for assessing radiographic success using two-dimensional imaging.

The main limitation of the current study lies in the relatively small sample size and the inability to analyze bone density around the apex in healing areas. Another limitation is the absence of microbiological swabs for all included samples to correlate the few observed failure cases with the nature of the bacterial flora present in the root canal system. Additionally, the studied sealers may be affected by the simple canal anatomy of the upper incisors and may not have the same effect on teeth with more complex canal anatomy. It is suggested to conduct studies with a similar design and a larger sample size on teeth with more complex canal anatomy using modern irrigation activation systems and modern bioceramic sealers compatible with warm vertical compaction technique using different assessment methods like assessing periapical lesion volume by MIMICs software to deepen comprehension of the healing dynamics of periapical lesions.

## Conclusion

In summary, this study demonstrates that large-sized periapical lesions have the potential to heal without surgical intervention. Successful treatment can be achieved using either bioceramic sealer with the single-cone technique or AH Plus sealer with lateral condensation technique with similar success rate up to 12 months follow up period.

## Data Availability

De-identified data are available upon reasonable request to the corresponding author.
